# Predicting clinical decline and conversion to Alzheimer’s disease or dementia using novel Elecsys Aβ(1–42), pTau and tTau CSF immunoassays

**DOI:** 10.1038/s41598-019-54204-z

**Published:** 2019-12-13

**Authors:** Kaj Blennow, Leslie M. Shaw, Erik Stomrud, Niklas Mattsson, Jon B. Toledo, Katharina Buck, Simone Wahl, Udo Eichenlaub, Valeria Lifke, Maryline Simon, John Q. Trojanowski, Oskar Hansson

**Affiliations:** 1 Clinical Neurochemistry Laboratory, Sahlgrenska University Hospital, Mölndal, Sweden; 2 Institute of Neuroscience and Physiology, Department of Psychiatry and Neurochemistry, The Sahlgrenska Academy at University of Gothenburg, Mölndal, Sweden; 3 Department of Pathology and Laboratory Medicine, University of Pennsylvania, Philadelphia, PA USA; 4 Clinical Memory Research Unit, Lund University, Malmö, Sweden; 5 Memory Clinic, Skåne University Hospital, Malmö, Sweden; 6 Wallenberg Center for Molecular Medicine, Lund University, Lund, Sweden; 7 Department of Neurology, Houston Methodist Hospital, Houston, TX USA; 8 Roche Diagnostics GmbH, Penzberg, Germany; 9 Roche Diagnostics International Ltd, Rotkreuz, Switzerland

**Keywords:** Neurological disorders, Alzheimer's disease

## Abstract

We evaluated the performance of CSF biomarkers for predicting risk of clinical decline and conversion to dementia in non-demented patients with cognitive symptoms. CSF samples from patients in two multicentre longitudinal studies (ADNI, n = 619; BioFINDER, n = 431) were analysed. Aβ(1–42), tTau and pTau CSF concentrations were measured using Elecsys CSF immunoassays, and tTau/Aβ(1–42) and pTau/Aβ(1–42) ratios calculated. Patients were classified as biomarker (BM)-positive or BM-negative at baseline. Ability of biomarkers to predict risk of clinical decline and conversion to AD/dementia was assessed using pre-established cut-offs for Aβ(1–42) and ratios; tTau and pTau cut-offs were determined. BM-positive patients showed greater clinical decline than BM-negative patients, demonstrated by greater decreases in MMSE scores (all biomarkers: –2.10 to –0.70). Risk of conversion to AD/dementia was higher in BM-positive patients (HR: 1.67 to 11.48). Performance of Tau/Aβ(1–42) ratios was superior to single biomarkers, and consistent even when using cut-offs derived in a different cohort. Optimal pTau and tTau cut-offs were approximately 27 pg/mL and 300 pg/mL in both BioFINDER and ADNI. Elecsys pTau/Aβ(1–42) and tTau/Aβ(1–42) are robust biomarkers for predicting risk of clinical decline and conversion to dementia in non-demented patients, and may support AD diagnosis in clinical practice.

## Introduction

Pathological processes underlying Alzheimer’s disease (AD) begin during a preclinical phase, often years before clinical symptoms associated with early stage disease^1^. Early diagnosis of AD and identification of disease progression are important for planning patient treatment and care. However, diagnosis at the mild cognitive impairment (MCI) stage, a known risk factor for progression, is challenging as: MCI does not always progress to dementia; dementia may be due to other causes; rates of progression vary; identifying individual conversion points is difficult^2,3^.

Amyloid (positron emission tomography [PET]) scanning is a Food and Drug Administration (FDA)-approved biomarker for supporting AD diagnosis^4^, with MCI patients showing evidence of amyloid pathology having a higher risk of clinical decline^5–7^. However, many amyloid-PET-positive patients remain cognitively normal for several years, highlighting the need for more robust biomarkers^8,9^. Recent efforts have focused on cerebrospinal fluid (CSF) biomarkers, and several studies have demonstrated the potential value of Aβ(1–42), phosphorylated Tau (181 P; pTau) and total Tau (tTau) biomarkers in MCI patients^9–15^.

Elecsys CSF immunoassays have been developed for measurement of Aβ(1–42), pTau and tTau, and have demonstrated excellent analytical performance, with high precision, good lot-to-lot comparability and low variability between and within laboratories^16–20^. Clinical evaluation in Alzheimer’s Disease Neuroimaging Initiative (ADNI) and BioFINDER studies also showed good concordance between measured CSF Aβ(1–42), ratios tTau/Aβ(1–42) and pTau/Aβ(1–42) and visual read outcomes of amyloid-PET^17^. We compare the performance of Aβ(1–42), pTau, tTau and ratios pTau/Aβ(1–42) and tTau/Aβ(1–42) for predicting the risk of clinical decline and conversion to AD or dementia in non-demented patients with cognitive symptoms.

## Methods

### Study populations

Individuals with MCI from ADNI and mild cognitive symptoms (MCS) from BioFINDER were included in the retrospective analyses, based on the following criteria: availability of a baseline Mini-Mental State Examination (MMSE) score; a baseline CSF sample; and a valid baseline measurement of the Elecsys biomarkers Aβ(1–42), pTau and tTau.

### ADNI

ADNI is an ongoing, longitudinal, multicentre study of volunteers with MCI or early AD, as well as cognitively normal healthy individuals enrolled at over 50 clinical centres, which started in 2004. Definitions of the participant classifications are presented below.

Normal cognition: MMSE scores between 24 and 30 (inclusive), a Clinical Dementia Rating (CDR) of 0, non-depressed, non-MCI and non-demented.

MCI: MMSE scores between 24 and 30 (inclusive), a memory complaint, have objective memory loss measured by education-adjusted scores on Wechsler Memory Scale Logical Memory II, a CDR of 0.5, absence of significant levels of impairment in other cognitive domains, essentially preserved activities of daily living and an absence of dementia.

Mild AD: MMSE scores between 20 and 26 (inclusive), CDR of 0.5 or 1.0 and meets National Institute of Neurological and Communicative Disorders and Stroke and the Alzheimer’s Disease and Related Disorders Association (NINCDS-ADRDA) criteria for probable AD.

Participants undergo annual examinations including magnetic resonance imaging (MRI) and amyloid-PET imaging, plasma and CSF sampling, as well as clinical and neuropsychological assessments. Further details are available at adni-info.org.

Overall, 619 of the total 872 patients (253 omitted due to missing baseline measurements) from the ADNI MCI population were included; 277 had early-MCI (criteria included memory function approximately 1.0 standard deviation [SD] below expected education-adjusted norms) and 342 had late-MCI (criteria included memory function approximately 1.5 SDs below expectation)^21^.

### BioFINDER

The control participants in BioFINDER were recruited from the population-based Malmö Diet Cancer Study^22^, and inclusion criteria were: aged > 60 years, MMSE score 28–30 at the screening visit, no cognitive symptoms and absence of MCI or dementia. Exclusion criteria were: presence of significant neurological or psychiatric disease, refusing lumbar puncture or MRI and significant alcohol or substance misuse. Subjects with MCS (i.e. either subjective cognitive decline [SCD] or MCI) were recruited consecutively at three memory clinics in southern Sweden. Inclusion criteria were: referral to the memory clinic due to cognitive symptoms experienced by the patient and/or an informant, criteria of any dementia disorder not fulfilled, MMSE score 24–30 and age 60–80 years. Exclusion criteria were: cognitive impairment that without doubt could be explained by a condition other than prodromal dementia, refusing lumbar puncture or neuropsychological investigation and current alcohol or substance misuse. The classification of MCS into SCD or MCI was based on a neuropsychological battery and the clinical assessment of a senior neuropsychologist as previously described^23^. AD diagnosis was confirmed by clinical evaluation and was based on the Diagnostic and Statistical Manual of Mental Disorders, 3rd Edition Revised (DSM-IIIR) criteria for dementia^24^ combined with the NINCDS-ADRDA criteria for AD^25^. Participants underwent bi-annual examinations including MRI, CSF and plasma sampling, and detailed clinical and neuropsychological assessments. Further details, including eligibility criteria, are available at BioFINDER.se. A total of 431 MCS patients from the BioFINDER population were included in the present analyses, and were classified into subgroups based on neuropsychological assessment: MCI (n = 233, including 172 patients with amnestic MCI), SCD (n = 191) or unknown SCD/MCI status (n = 7).

### Ethical approval and informed consent

The final version of the protocol was approved by the Copernicus Group Independent Review Board and Regional Ethics Review Board in Lund. All procedures performed in studies involving human participants were in accordance with the ethical standards of the institutional and/or national research committee and with the 1964 Helsinki Declaration and its later amendments or comparable ethical standards. Written informed consent was obtained from all patients who participated in ADNI and BioFINDER.

### Biomarker measurements

CSF concentrations of Aβ(1–42), pTau and tTau were measured using Elecsys CSF immunoassays on a cobas e 601 analyser at the University of Pennsylvania (ADNI) and the University of Gothenburg (BioFINDER).

### Pre-specified cut-offs for CSF Aβ(1–42), pTau/Aβ(1–42) and tTau/Aβ(1–42)

Based on BioFINDER data, cut-off values for Aβ(1–42), pTau/Aβ(1–42) and tTau/Aβ(1–42) have previously been determined for concordance between CSF biomarkers and visual read of amyloid-PET images^17^. The previously derived cut-off values were: Aβ(1–42), 1,100 pg/mL; pTau/Aβ(1–42), 0.022; tTau/Aβ(1–42), 0.26. Similarly, for ADNI, the cut-offs optimised for concordance of CSF biomarkers with amyloid-PET visual read were: Aβ(1–42), 977 pg/mL; pTau/Aβ(1–42), 0.025; tTau/Aβ(1–42), 0.27^17^. As a sensitivity analysis, ADNI analyses were also performed using cut-offs derived from BioFINDER and adjusted for pre-analytical handling in ADNI: Aβ(1–42), 880 pg/mL; pTau/Aβ(1–42), 0.028; tTau/Aβ(1–42), 0.33, as previously described^17^.

### Statistical analyses

Analyses were conducted using SAS version 9.4 and R version 3.4.0.

### Derivation of cut-offs for CSF pTau and tTau

To derive cut-offs for the assessment of clinical decline by single tau biomarkers (pTau and tTau), progression analyses were performed across a grid of cut-offs (in 2.5% steps) in ADNI. Based on findings for different models (as specified below), and outcomes evaluated across the grid, cut-offs were derived using visual assessment that provided a good separation between MCI patients with a higher *versus* lower risk of clinical decline. The ability of biomarker status (based on the selected cut-offs) to predict risk of clinical decline was evaluated in the BioFINDER population.

As a sensitivity analysis, cut-offs were derived based on concordance (Youden-index optimisation) with the outcome AD *versus* cognitively normal controls in both studies.

### Mixed-effects modeling

Based on biomarker status (as specified by the cut-offs), CSF samples were classified as biomarker-positive (BM-positive) or biomarker-negative (BM-negative). MMSE scores from visits at baseline, 12 and 24 months for BioFINDER and from visits at baseline, 6, 12 and 24 months for ADNI were evaluated as the main outcome measure. Clinical Dementia Rating Scale Sum of Boxes (CDR-SB; ADNI), Functional Activities Questionnaire (FAQ; ADNI and BioFINDER) and Alzheimer’s Disease Assessment Scale-cognitive (ADAS-cog; ADNI) were also evaluated as outcome measures.

Prediction of change in clinical score based on biomarker status was analysed using linear mixed-effects regression models, including random effects (random intercepts) for the patient and fixed effects for biomarker test result at baseline (BM-negative and BM-positive), visit (categorical), baseline clinical score (continuous), interaction between visit and baseline clinical score, interaction between visit and biomarker test result and the adjustment covariates age, sex and years of education (with [data not shown] and without adjustment for *APOE*ε4 allele status). The model was fitted using restricted maximum likelihood estimation and the Satterthwaite approximation for the degrees of freedom. The model was used to evaluate the following three effects: change in clinical score from baseline to 24 months in BM-negative patients; change in clinical score from baseline to 24 months in BM-positive patients; difference in clinical scores from baseline to 24 months between BM-positive and BM-negative patients.

### Time-to-event modeling

Time-to-event analyses were performed for the outcome time-to-dementia diagnosis in the ADNI MCI and BioFINDER MCS populations (6 years' follow-up). In the BioFINDER MCS population, time to AD diagnosis was also assessed; this was not assessed in ADNI, as most subjects progressed to AD. Cox proportional hazards models were fitted with covariate biomarker status (BM-negative and BM-positive) adjusted for age, sex, years of education, baseline MMSE and baseline CDR-SB (ADNI). Hazard ratio (HR) estimates with 95% confidence intervals (CIs) were obtained and Kaplan-Meier curves estimated according to biomarker status.

### Multi-marker modeling

Mixed-effects and time-to-event analyses were performed to evaluate the contribution of tau, in addition to amyloid biomarkers, to the prediction of risk of clinical decline and conversion to dementia, when tau was combined with markers of amyloid pathology: Aβ(1–42), Tau/Aβ(1–42) ratios or amyloid-PET. The evaluation was performed using linear mixed-effects and Cox proportional hazards models as described above, and four-categorical variables were defined as: amyloid + |Tau + , amyloid –|Tau + , amyloid + |Tau –, amyloid –|Tau –. Likelihood ratio tests (LRTs) were used to assess the contribution of tau in the models.

## Results

### Study populations

Baseline characteristics for ADNI (MCI) and BioFINDER (MCS) populations are presented in Table 1. Age, baseline MMSE and *APOE*ε4 genotype were broadly similar between the ADNI and BioFINDER populations, but key differences included lower measured baseline Aβ(1–42) concentrations (962 *versus* 1,142 pg/mL) and baseline FAQ score (3.06 *versus* 5.67), and higher proportion of patients with a first-degree family history of dementia (57% *versus* 41%).

**Table 1:** Baseline characteristics for the ADNI MCI and the BioFINDER MCS populations and subcohorts.

Characteristic		ADNI			BioFINDER		
		Overall (N = 619)	EMCI (n = 277)	LMCI (n = 342)	Overall (N = 431)	SCD (n = 191)	MCI (n = 233)
Cohort, N (%)	ADNI1	187 (30.21)	0	187 (54.68)	—	—	—
	ADNIGO	117 (18.90)	117 (42.24)	0	—	—	—
	ADNI2	315 (50.89)	160 (57.76)	155 (45.32)	—	—	—
Age [years], mean (SD)		72 (7.6)	71 (7.4)	73 (7.6)	70 (5.6)	70 (5.7)	71 (5.5)
Sex, male, N (%)		364 (58.80)	155 (55.96)	209 (61.11)	233 (54.06)	89 (46.60)	142 (60.94)
*APOE*ε4 genotype grouped (number of risk alleles), N (%)	0	314 (50.73)	160 (57.76)	154 (45.03)	234 (54.55)	114 (60.32)	116 (49.79)
	1	239 (38.61)	97 (35.02)	142 (41.52)	151 (35.20)	62 (32.80)	86 (36.91)
	2	66 (10.66)	20 (7.22)	46 (13.45)	44 (10.26)	13 (6.88)	31 (13.30)
Education [years], mean (SD)		16.09 (2.76)	15.95 (2.65)	16.20 (2.83)	11.8 (3.48)	12.5 (3.56)	11.2 (3.28)
Family history of dementia (first degree), N (%)	Yes	353 (57.03)	167 (60.29)	186 (54.39)	169 (40.92)	79 (42.70)	89 (40.09)
	No	260 (42.00)	106 (38.27)	154 (45.03)	244 (59.08)	106 (57.30)	133 (59.91)
Family history of AD (first degree), N (%)	Yes	214 (34.57)	108 (38.99)	106 (30.99)	—	—	—
	No	138 (22.29)	25 (9.03)	113 (33.04)	—	—	—
Baseline clinical score, mean (SD)	CDR-SB	1.48 (0.89)	1.29 (0.77)	1.64 (0.94)	—	—	—
	MMSE	27.74 (1.81)	28.35 (1.58)	27.24 (1.83)	27.7 (1.81)	28.5 (1.40)	27.1 (1.84)
	FAQ	3.06 (4.02)	2.10 (3.20)	3.83 (4.43)	5.67 (5.04)	3.90 (4.39)	6.93 (4.99)
	ADAS-cog	16.11 (6.91)	12.70 (5.39)	18.87 (6.77)	—	—	—
Baseline biomarker measurement, mean (SD; pg/mL)	Aβ(1–42)	962.0 (437.0)	1,093 (438.9)	855.7 (406.1)	1,142 (450.5)	1,272 (431.9)	1,038 (439.5)
	pTau	27.83 (15.01)	24.25 (13.69)	30.72 (15.42)	23.61 (12.79)	21.62 (11.24)	24.94 (13.55)
	tTau	287.0 (134.6)	256.4 (121.7)	311.8 (139.5)	262.9 (119.2)	242.6 (101.9)	276.8 (127.9)

ADNI, Alzheimer’s Disease Neuroimaging Initiative; MCI, mild cognitive impairment; MCS, mild cognitive symptoms; EMCI, early mild cognitive impairment; LMCI, late mild cognitive impairment; SCD, subjective cognitive decline; SD, standard deviation; AD, Alzheimer’s disease; CDR-SB, Clinical Dementia Rating Scale Sum of Boxes; MMSE, Mini-Mental State Examination; FAQ, Functional Activities Questionnaire; ADAS-cog, Alzheimer’s Disease Assessment Scale-cognitive; pTau, phosphorylated Tau; tTau, total Tau.

### Derivation of cut-offs for CSF pTau and tTau

Patient classification as BM-positive *versus* BM-negative using single tau biomarker cut-offs of 27 pg/mL (pTau) and 300 pg/mL (tTau) provided good separation between patients with higher *versus* lower risk of clinical decline, and these values were therefore selected for evaluation. The selected cut-offs showed robust separation of BM-positive and BM-negative patients when clinical decline was based on change in the clinical scores MMSE, CDR-SB (ADNI only), ADAS-cog (ADNI only) and FAQ, or dementia diagnosis in ADNI (Supplementary Figs. S1–S3).

In a sensitivity analysis, single tau cut-offs were optimised for identification of AD patients *versus* normal controls in the BioFINDER and ADNI populations. Cut-offs identified were 28 pg/mL (pTau) and 307 pg/mL (tTau) in BioFINDER, and 24 pg/mL (pTau) and 266 pg/mL (tTau) in ADNI. Derived cut-offs for each study were similar and demonstrated a similar performance to the original cut-offs of 27 pg/mL (pTau) and 300 pg/mL (tTau) in both study populations, thus confirming the robustness of the chosen cut-offs (Supplementary Figs. S1–S3).

### CSF biomarkers as predictors of clinical decline

**Figure 1:**
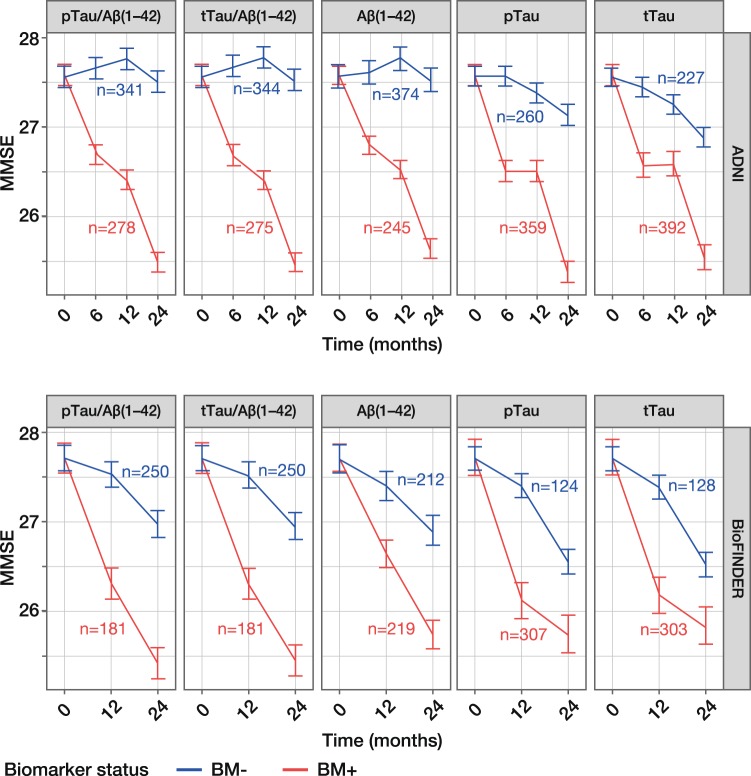
Model-derived time-course plots of MMSE score (24 months) according to CSF biomarker status. Least square-means with SEs are presented for the ADNI MCI and BioFINDER MCS populations. ADNI (upper panel) cut-offs: pTau/Aβ(1–42), 0.025; tTau/Aβ(1–42), 0.27; Aβ(1–42), 977 pg/mL. BioFINDER (lower panel) cut-offs: pTau/Aβ(1–42), 0.022; tTau/Aβ(1–42), 0.26; Aβ(1–42), 1,100 pg/mL. pTau and tTau cut-offs of 27 pg/mL and 300 pg/mL, respectively, were used in both cohorts. Analyses shown with adjustment for age, sex, years of education but without adjustment for *APOE*ε4 status; number of patients in each biomarker group at baseline is presented. MMSE, Mini-Mental State Examination; CSF, cerebrospinal fluid; pTau, phosphorylated Tau; tTau, total Tau; ADNI, Alzheimer’s Disease Neuroimaging Initiative; BM–, biomarker-negative; BM + , biomarker-positive; SE, standard error; MCI, mild cognitive impairment; MCS, mild cognitive symptoms.

MMSE scores for BM-negative patients remained stable from baseline to 24 months, with a mean change of –1.20 to –0.04 across all five biomarkers in the ADNI and BioFINDER populations (Table 2). In contrast, MMSE scores for BM-positive patients decreased steadily, with a mean change of –2.31 to –1.90, indicating that cognitive decline was greater among BM-positive compared with BM-negative patients. This trend was evident in both ADNI and BioFINDER populations and for all five biomarkers (Table 2; Fig. 1); however, amongst BM-negative patients, mean changes in MMSE score were slightly lower in ADNI (–0.68 to –0.04) compared with BioFINDER (–1.20 to –0.74).

**Table 2:** Prediction of clinical decline (24 months) assessed by MMSE scores, according to CSF biomarker status.

Biomarker	Change in score, BM-positive, estimate (95% CI)		Change in score, BM-negative, estimate (95% CI)		Difference between change in score, BM-negative and BM-positive, estimate (95% CI)	
	ADNI	BioFINDER	ADNI	BioFINDER	ADNI	BioFINDER
pTau/Aβ(1–42)	–2.13 (–2.39 to –1.87)	–2.31 (–2.73 to –1.89)	–0.05 (–0.34 to 0.24)	–0.74 (–1.10 to –0.38)	–2.08 (–2.47 to –1.68)	–1.57 (–2.14 to –1.01)
tTau/Aβ(1–42)	–2.13 (–2.39 to –1.88)	–2.28 (–2.69 to –1.86)	–0.04 (–0.33 to 0.25)	–0.77 (–1.12 to –0.41)	–2.10 (–2.49 to –1.71)	–1.51 (–2.07 to –0.95)
Aβ(1–42)	–1.96 (–2.21 to –1.71)	–1.99 (–2.37 to –1.62)	–0.04 (–0.34 to 0.27)	–0.81 (–1.19 to –0.42)	–1.92 (–2.32 to –1.53)	–1.19 (–1.74 to –0.64)
pTau	–2.23 (–2.53 to –1.94)	–1.99 (–2.49 to –1.49)	–0.43 (–0.69 to –0.18)	–1.16 (–1.48 to –0.84)	–1.80 (–2.20 to –1.40)	–0.83 (–1.44 to –0.22)
tTau	–2.07 (–2.39 to –1.75)	–1.90 (–2.39 to –1.40)	–0.68 (–0.93 to –0.43)	–1.20 (–1.52 to –0.87)	–1.40 (–1.81 to –0.99)	–0.70 (–1.30 to –0.10)

Based on PET-optimised cut-offs for Aβ(1–42), pTau/Aβ(1–42) and tTau/Aβ(1–42). Analyses shown with adjustment for age, sex, years of education but without adjustment for *APOE*ε4 status. Change in score calculated from baseline to 24 months in the ADNI MCI and BioFINDER MCS populations. MMSE, Mini-Mental State Examination; CSF, cerebrospinal fluid; BM, biomarker; CI, confidence interval; ADNI, Alzheimer’s Disease Neuroimaging Initiative; pTau, phosphorylated Tau; tTau, total Tau; PET, positron emission tomography; MCI, mild cognitive impairment; MCS, mild cognitive symptoms.

The difference in change in MMSE score between BM-negative and BM-positive patients ranged from –2.10 to –0.70 across all five biomarkers in both populations, with upper 95% confidence limits of < 0. The difference between BM-positive and BM-negative patients was a little more pronounced in ADNI (–2.10 to –1.40) than in BioFINDER (1.57 to –0.70) (Table 2; Fig. 1); the smaller between-group difference in BioFINDER reflects the greater change in MMSE score in BM-negative patients described above. Estimates for all covariates can be found in Supplementary Table S1. Additional analyses based on FAQ, CDR-SB (ADNI only) and ADAS-cog (ADNI only) clinical scores also showed good separation between BM-negative and BM-positive patients, indicating greater clinical decline among BM-positive patients (Supplementary Table S2; Supplementary Fig. S4). Sensitivity analyses, to evaluate the robustness of the data, show that risk of clinical decline was accurately predicted in the ADNI cohort even when using cut-offs derived from BioFINDER and adjusted to account for differences in pre-analytical handling in ADNI (Supplementary Table S3; Supplementary Fig. S5).

When comparing the performance of each biomarker for predicting risk of clinical decline, the Tau/Aβ(1–42) ratios were superior to single biomarkers, as demonstrated by the greater difference in MMSE scores between BM-negative and BM-positive patients, i.e. –2.10 for tTau/Aβ(1–42) *versus* –1.40 for tTau in ADNI (Table 2). When comparing the performance of different cut-offs, separation between MMSE scores for BM-negative and BM-positive patients was robust, and the PET-optimised cut-offs were not substantially outperformed by any of the other cut-offs analysed; findings were consistent for clinical scores CDR-SB, FAQ and ADAS-cog (Supplementary Figs. S1 and S2).

### CSF biomarkers for prediction of conversion to dementia or AD

**Figure 2:**
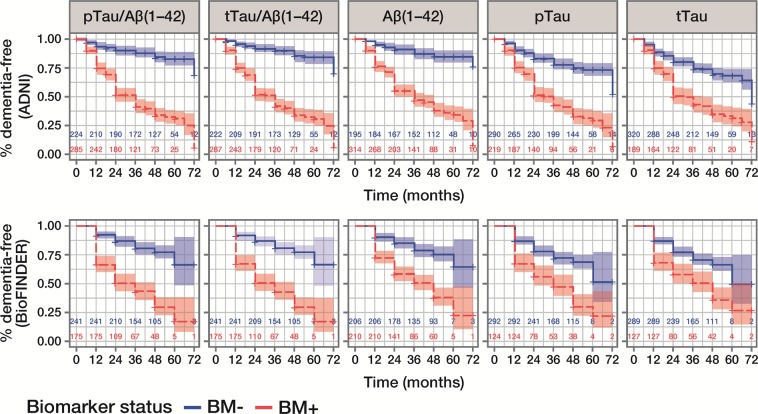
Kaplan-Meier curves for outcome all-cause dementia diagnosis within 6 years, according to CSF biomarker status. Data are presented for the ADNI MCI and BioFINDER MCS populations; number of patients in each biomarker group at each time point is presented. ADNI (upper panel) cut-offs: pTau/Aβ(1–42), 0.025; tTau/Aβ(1–42), 0.27; Aβ(1–42), 977 pg/mL. BioFINDER (lower panel) cut-offs: pTau/Aβ(1–42), 0.022; tTau/Aβ(1–42), 0.26; Aβ(1–42), 1,100 pg/mL. pTau and tTau cut-offs of 27 pg/mL and 300 pg/mL, respectively, were used in both cohorts. CSF, cerebrospinal fluid; ADNI, Alzheimer’s Disease Neuroimaging Initiative; pTau, phosphorylated Tau; tTau, total Tau; BM–, biomarker-negative; BM + , biomarker-positive; MCI, mild cognitive impairment; MCS, mild cognitive symptoms.

CSF biomarker status at baseline identified patients with a higher (BM-positive) *versus* lower (BM-negative) risk of conversion to dementia within 6 years, as demonstrated by good separation on Kaplan-Meier curves (Fig. 2). HRs for conversion to dementia were highest for pTau/Aβ(1–42) and tTau/Aβ(1–42), and lowest for pTau and tTau (Table 3). Although HRs for conversion to all-cause dementia were lower in BioFINDER than in ADNI, exploration of conversion to AD dementia in BioFINDER showed larger HRs; the greatest differences were observed for pTau/Aβ(1–42) (HR 11.48 *versus* 3.38) and tTau/Aβ(1–42) (HR 10.31 *versus* 3.38; Table 3).

**Table 3:** Hazard ratios (Cox proportional regression) for conversion to dementia or AD, by CSF biomarker status.

Biomarker	Hazard ratio (95% CI)		
	ADNI	BioFINDER	
	Dementia	Dementia	AD
pTau/Aβ(1–42)	4.76 (3.22–7.04)	3.38 (2.35–4.87)	11.48 (6.04–21.81)
tTau/Aβ(1–42)	5.20 (3.48–7.78)	3.38 (2.35–4.86)	10.31 (5.55–19.13)
Aβ(1–42)	4.41 (2.89–6.72)	2.63 (1.83–3.78)	6.00 (3.38–10.65)
pTau	2.73 (2.02–3.70)	1.94 (1.39–2.72)	3.86 (2.51–5.95)
tTau	2.12 (1.59–2.84)	1.67 (1.20–2.33)	3.00 (1.98–4.55)

Analyses shown with adjustment for age, sex, years of education, baseline MMSE score, baseline CDR-SB score (ADNI only), but without adjustment for *APOE*ε4 status. Data presented for ADNI MCI and BioFINDER MCS populations. AD, Alzheimer’s disease; CSF, cerebrospinal fluid; CI, confidence interval; ADNI, Alzheimer’s Disease Neuroimaging Initiative; pTau, phosphorylated Tau; tTau, total Tau; MMSE, Mini-Mental State Examination; CDR-SB, Clinical Dementia Rating Sum of Boxes; MCI, mild cognitive impairment; MCS, mild cognitive symptoms.

When comparing the performance of different cut-offs across a grid, results were robust, and cut-offs derived in BioFINDER and adjusted for differences in pre-analytical handling procedure in ADNI showed similar results to those based on the PET-optimised cut-offs, for all clinical scores (Supplementary Fig. S3).

### Contribution of single tau biomarkers when combined with biomarkers of amyloid status for the prediction of risk of clinical decline and conversion to dementia

**Figure 3:**
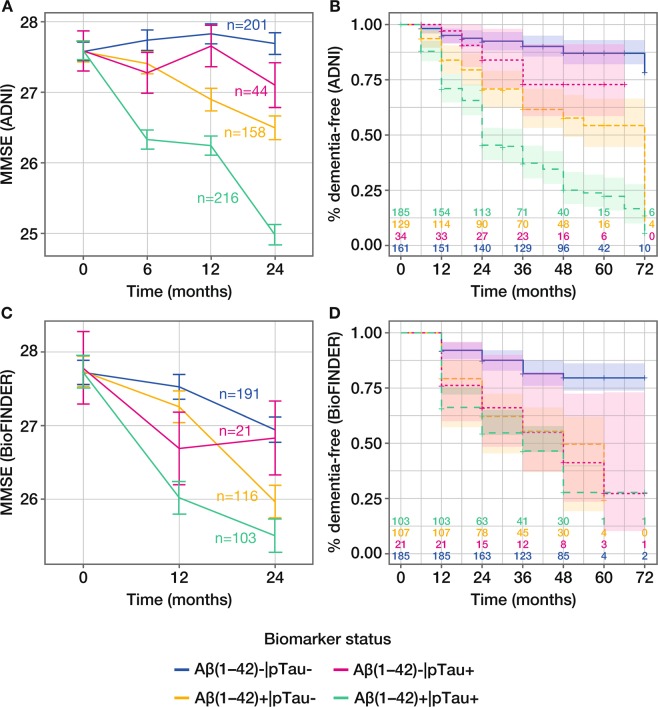
Evaluation of pTau and Aβ(1–42) for predicting clinical decline and conversion to all-cause dementia. Model-derived time-course plot (least square-means with SE) of clinical decline assessed by change in MMSE score from baseline to 24 months in the (**A**) ADNI MCI and (**c**) BioFINDER MCS populations; adjustment for age, sex, years of education but without adjustment for *APOE*ε4 status; number of patients in each biomarker group at baseline is presented. Kaplan-Meier curves of outcome dementia diagnosis within 6 years in the (**B**) ADNI MCI and (**D**) BioFINDER MCS populations; number of patients in each biomarker group at each time point is presented. pTau, phosphorylated Tau; MMSE, Mini-Mental State Examination; ADNI, Alzheimer’s Disease Neuroimaging Initiative; SE, standard error; MCI, mild cognitive impairment; MCS, mild cognitive symptoms.

Mixed-effects model estimates of differences in clinical decline from baseline to 24 months, based on MMSE score, were greatest for patients who were positive for both biomarkers compared with patients who were negative for both biomarkers (reference) across all biomarkers in both ADNI and BioFINDER populations. When considering amyloid status defined by Aβ(1–42), the mixed-effects model estimate of the difference between Aβ(1–42) + |pTau + and Aβ(1–42)–|pTau– was –2.72 in ADNI and –1.46 in BioFINDER, with clear separation according to biomarker status demonstrated in model-derived time-course plots (Table 4; Fig. 3A, C). In both cases, the contribution of pTau was significant, with P_LRT_ ≤ 0.05 (Table 4). The contribution of pTau was also significant when added to amyloid status defined by pTau/Aβ(1–42) or visual PET read-out in the ADNI population, but not in the smaller BioFINDER population (Table 4; Supplementary Figs. S6 and S7). Results were also similar for the contribution of tTau (Table 4).

**Table 4:** Contribution of tau biomarkers to prediction of clinical decline assessed by MMSE score (24 months).

	Biomarker	Mixed-model estimate (95% CI) of difference in clinical decline (24 months) *versus* reference, BM–/BM–			LRT P value	Hazard ratio estimate (95% CI) for outcome dementia within 6 years			LRT P value
		BM1–|BM2 +	BM1 + |BM2–	BM1 + |BM2 +		BM1–|BM2+	BM1+|BM2–	BM1+|BM2+	
ADNI	Aβ(1–42)|pTau	–0.60 (–1.38 to 0.19)	–1.19 (–1.69 to –0.69)	–2.72 (–3.20 to –2.24)	<0.0001^a^	2.05 (0.88 to 4.76)	3.46 (1.99 to 6.01)	6.61 (3.99 to 10.96)	0.0001^a^
	pTau/Aβ(1–42)|pTau	0.02 (–1.07 to 1.11)	–1.34 (–1.90 to –0.79)	–2.40 (–2.84 to –1.96)	0.001^b^	0.46 (0.06 to 3.36)	3.56 (2.19 to 5.80)	5.02 (3.34 to 7.54)	0.117^b^
	Visual PET|pTau	0.02 (–0.87 to 0.92)	–0.82 (–1.38 to –0.26)	–2.33 (–2.81 to –1.85)	<0.0001^c^	0.77 (0.10 to 5.89)	2.97 (1.46 to 6.04)	7.25 (4.02 to 13.11)	0.002^c^
	Aβ(1–42)|tTau	–0.53 (–1.30 to 0.25)	–1.53 (–2.01 to –1.05)	–2.60 (–3.10 to –2.10)	<0.0001^d^	1.73 (0.72 to 4.14)	3.94 (2.34 to 6.62)	6.09 (3.69 to 10.07)	0.013^d^
	tTau/Aβ(1–42)|tTau	0.11 (–0.86 to 1.08)	–1.73 (–2.23 to –1.23)	–2.35 (–2.81 to –1.89)	0.001^e^	0.44 (0.06 to 3.28)	4.70 (2.98 to 7.39)	5.19 (3.39 to 7.96)	0.551^e^
	Visual PET|tTau	–0.07 (–0.91 to 0.76)	–1.00 (–1.51 to –0.49)	–2.54 (–3.05 to –2.03)	<0.0001^f^	0.76 (0.10 to 5.88)	3.86 (2.03 to 7.35)	7.53 (4.10 to 13.82)	0.012 ^f^
BioFINDER	Aβ(1–42)|pTau	–0.18 (–1.45 to 1.10)	–0.99 (–1.65 to –0.33)	–1.46 (–2.15 to –0.76)	0.002^a^	2.17 (1.06 to 4.45)	2.71 (1.72 to 4.27)	3.50 (2.25 to 5.45)	0.060^a^
	pTau/Aβ(1–42)|pTau	0.26 (–1.40 to 1.93)	–1.75 (–2.53 to –0.98)	–1.44 (–2.09 to –0.78)	0.100^b^	1.12 (0.38 to 3.27)	3.22 (1.82 to 5.71)	3.90 (2.39 to 6.35)	0.926^b^
	Visual PET|pTau	–0.79 (–2.20 to 0.63)	–2.24 (–3.19 to –1.30)	–1.74 (–2.51 to –0.96)	0.058^c^	1.12 (0.35 to 3.65)	3.57 (2.27 to 5.63)	3.33 (2.23 to 4.97)	0.743^c^
	Aβ(1–42)|tTau	–0.19 (–1.32 to 0.94)	–1.05 (–1.71 to –0.38)	–1.42 (–2.13 to –0.71)	0.002^d^	1.88 (0.95 to 3.73)	2.85 (1.80 to 4.50)	3.40 (2.15 to 5.37)	0.146^d^
	tTau/Aβ(1–42)|tTau	0.08 (–1.26 to 1.41)	–1.71 (–2.47 to –0.94)	–1.37 (–2.03 to –0.71)	0.110^e^	1.36 (0.54 to 3.42)	3.36 (1.87 to 6.04)	4.04 (2.46 to 6.65)	0.768^e^
	Visual PET|tTau	–0.76 (–2.05 to 0.53)	–2.24 (–3.20 to –1.28)	–1.78 (–2.55 to –1.00)	0.056^f^	1.10 (0.43 to 2.82)	3.73 (2.38 to 5.84)	3.24 (2.15 to 4.89)	0.623 ^f^

Analyses shown with adjustment for age, sex, years of education but without adjustment for *APOE*ε4 status. Hazard ratio data also adjusted for baseline MMSE score (ADNI and BioFINDER) and baseline CDR-SB score (ADNI only). LRTs were used to assess the contribution of tau when combined with Aβ(1–42), Tau/Aβ(1–42) ratios or amyloid-PET, based on comparison of different four-categorical mixed models and Cox regression models. Data presented for ADNI MCI and BioFINDER MCS populations. LRT comparison: ^a^Aβ(1–42) *versus* Aβ(1–42)|pTau; ^b^pTau/Aβ(1–42) *versus* pTau/Aβ(1–42)|pTau; ^c^Visual PET *versus* visual PET|pTau; ^d^Aβ(1–42) *versus* Aβ(1–42)|tTau; ^e^tTau/Aβ(1–42) *versus* tTau/Aβ(1–42)|tTau; ^f^Visual PET *versus* visual PET|tTau. MMSE, Mini-Mental State Examination; CI, confidence interval; BM–, biomarker-negative; BM + , biomarker-positive; LRT, likelihood ratio test; ADNI, Alzheimer’s Disease Neuroimaging Initiative; pTau, phosphorylated Tau; PET, positron emission tomography; tTau, total Tau; CDR-SB, Clinical Dementia Rating Scale Sum of Boxes; MCI, mild cognitive impairment; MCS, mild cognitive symptoms.

Time-to-event modelling, considering amyloid status defined by Aβ(1–42) in combination with pTau, showed clear separation according to biomarker status for analysis of conversion to all-cause dementia in ADNI, and to a lesser extent in BioFINDER (Fig. 3B, D). Corresponding HRs were consistently highest for amyloid + |Tau + compared with the reference amyloid–|Tau– and confirmed the contribution of tau for predicting conversion to dementia (Table 4).

## Discussion

Our findings show that Aβ(1–42), pTau and tTau are promising CSF biomarkers for predicting the risk of clinical decline and conversion to AD or dementia in patients with MCI/MCS, using novel Elecsys immunoassays. Tau/Aβ(1–42) ratios demonstrated superior performance to the single CSF biomarkers Aβ(1–42), pTau and tTau. Cut-off values for single tau biomarkers were also derived and validated.

The ability to diagnose AD early in the disease course and identify disease progression is of utmost importance for planning patient treatment and care. Robust biomarkers that can accurately assess clinical decline are therefore needed to support clinical assessment of patients in practice and the evaluation of potentially disease-modifying drugs in trials. This study demonstrates that classification of MCI/MCS patients as BM-positive or BM-negative according to CSF biomarkers pTau/Aβ(1–42), tTau/Aβ(1–42), Aβ(1–42), pTau or tTau can distinguish between those who are at higher *versus* lower risk of clinical decline, based on the change in clinical scores over 24 months. In addition, time-to-event analyses show that these biomarkers can predict the risk of conversion to dementia within 6 years according to BM-negative *versus* BM-positive status at baseline. Importantly, we validated the ability to predict a patient’s risk of clinical decline using pTau/Aβ(1–42), tTau/Aβ(1–42) and Aβ(1–42) cut-offs previously derived for PET concordance.

Previous studies of CSF biomarkers Aβ(1–42), pTau and tTau have reported encouraging evidence for their utility as predictors of clinical decline^11,26,27^. In our comprehensive evaluation of five CSF biomarkers across four clinical scoring algorithms, we demonstrate the consistently superior performance of the Tau/Aβ(1–42) ratios compared with single biomarkers for prediction of clinical decline and conversion to dementia. Specifically, pTau/Aβ(1–42) demonstrated the best performance for prediction of clinical decline in MCI patients over 24 months. These data suggest that Tau/Aβ(1–42) ratios are the most sensitive and specific of the AD CSF biomarkers currently under investigation, a finding supported by previous studies^10,12,28^. Superior performance with both pTau/Aβ(1–42) and tTau/Aβ(1–42) compared with Aβ(1–42) observed in our study may relate to the extended degree of neuronal dysfunction and death, induced by tau hyper-phosphorylation and aggregation^29,30^. It could also be linked to improved amyloid-PET-concordance of Tau/Aβ(1–42) ratios compared with Aβ(1–42), pTau and tTau^31–33^. This seems to reflect AD pathology, as amyloid-PET imaging mostly detects neuritic plaques, containing tau and Aβ^29^.

For the first time, we report cut-offs for single pTau and tTau CSF biomarkers; these cut-offs were derived using the ADNI population and then validated in BioFINDER. Our approach of optimising CSF biomarker cut-offs for concordance with amyloid-PET status, as previously used for derivation of Aβ(1–42) and Tau/Aβ(1–42) ratio cut-offs, is therefore newly validated as a method for determining cut-offs for clinical decline analyses. Of note, the optimal cut-offs for pTau and tTau were similar in both cohorts even though CSF was collected using different protocols and the Elecsys analyses were performed in different laboratories, demonstrating the robustness and transferability of data generated with the Elecsys pTau and tTau CSF assays.

Analyses conducted based on tau status added to an Aβ assessment, such as Aβ(1–42), Tau/Aβ(1–42) or visual PET, may closely reflect clinical practice and provide insight into performance differences between biomarkers^34^. Using the newly established single tau cut-offs, we showed that the benefit of tau in addition to Aβ(1–42) is consistently significant across two study populations. However, the added benefit of tau in addition to Tau/Aβ(1–42) or PET was less compelling. This may be because Aβ and combined Aβ/tau pathology are strong predictors of clinical decline and conversion to AD, whereas tau is associated with other pathologies, and is therefore not an ideal single marker for the prediction of specific dementia types. Further, the effect of tau in addition to Tau/Aβ(1–42) may be minimal because tau is already present in the ratio. Small sample sizes limit the conclusions that can be drawn. Our findings are, however, consistent with other studies where tau showed clinical value in combination with Aβ(1–42)^34^, and Aβ(1–42) only predicted conversion to AD dementia when combined with pTau as a ratio^10,35^. Such findings are consistent with the hypothesis that as tau pathology emerges with pre-existing amyloid pathology, the overall rate of disease progression increases^35^.

Notably, all biomarkers predicted progression more strongly in ADNI compared with BioFINDER, which may reflect the smaller sample size and differences in population characteristics. ADNI was developed to simulate an AD clinical trial, which is reflected in the enrolment criteria and may have resulted in a more select AD population compared with BioFINDER, by including fewer patients with other forms of dementia or any significant neurological disease other than AD. This may also account for the stronger performance of the CSF biomarkers when used to predict conversion to AD, rather than all-cause dementia, in BioFINDER. In BioFINDER, although most dementia cases are due to AD, many patients developed other forms of dementia, consistent with post-mortem studies comprising patients with MCI at baseline^36^. Similarly, although AD may be the primary diagnosis in ADNI patients, many patients had more than one co-pathology such as Lewy body pathology and TDP-43 deposits (observed in approximately 42% and 21% of patients, respectively)^37,38^.

Strengths of this study include the comprehensive analysis of samples from two large, international cohorts, including many follow-up visits, thus improving the reliability of the results. The Elecsys immunoassays have an excellent analytical performance, therefore enabling accurate and precise measurement of CSF biomarker concentrations between and within laboratories. This is demonstrated in both the consistency of cut-offs for pTau and tTau, when derived from CSF samples collected from two different cohorts and analysed at different laboratories, and the ability of Tau/Aβ(1–42) ratios to accurately predict risk of cognitive decline in one cohort (ADNI) even when using cut-offs established in another cohort (BioFINDER). Our approach of optimising CSF Tau/Aβ(1–42) ratio cut-offs for concordance with amyloid-PET status also contributed to the consistency of these analyses. Findings were also consistent between both continuous clinical scores as outcomes and time-to-event analyses.

This study provides valuable data supporting the potential benefits of CSF biomarker assessment as a robust and accurate alternative to imaging techniques. Advantages of CSF biomarkers over imaging techniques include lower cost and the opportunity to detect other pathologies by the same procedure, for example analysing other CSF components such as neurofilament light chain and neurogranin. Plasma-based assays are also in development, and could provide a less invasive means of assessing patients with cognitive symptoms and suspected AD in primary care settings^39^. However, measurement of AD biomarkers, e.g. tau protein and Aβ(1–42), in blood samples may face analytical challenges due to their low abundance relative to the very high levels of plasma proteins, resulting in matrix interference, as well as possible biological confounders such as expression of these proteins in peripheral tissue with release into plasma^40^. Greater utilisation of CSF biomarkers in clinical trials could aid identification of appropriate patients most likely to benefit from potentially disease-modifying drugs and help to assess their efficacy^11,13,41^. Elecsys CSF assays also offer the benefit of minimising potential inter-observer variability that can occur with imaging.

## Supplementary information

Supplement

## Data Availability

ADNI data are available at http://adni.loni.usc.edu/data-samples/access-data/. For the BioFINDER study, anonymised data is available upon request from any qualified investigator for the sole purpose of replicating procedures and results presented in the article, subject to data transfer aligning with EU legislation on the General Data Protection Regulation.

## References

[1] Sperling RA (2011). Toward defining the preclinical stages of Alzheimer’s disease: recommendations from the National Institute on Aging-Alzheimer’s Association workgroups on diagnostic guidelines for Alzheimer’s disease. Alzheimers Dement..

[2] Albert MS (2011). The diagnosis of mild cognitive impairment due to Alzheimer’s disease: recommendations from the National Institute on Aging-Alzheimer’s Association workgroups on diagnostic guidelines for Alzheimer’s disease. Alzheimers Dement..

[3] Petersen RC (2004). Mild cognitive impairment as a diagnostic entity. J. Intern. Med..

[4] Apostolova LG (2016). Critical review of the appropriate use criteria for amyloid imaging: effect on diagnosis and patient care. Alzheimers Dement..

[5] Doraiswamy PM (2014). Florbetapir F 18 amyloid PET and 36-month cognitive decline: a prospective multicenter study. Mol. Psychiatry.

[6] Nordberg A (2013). A European multicentre PET study of fibrillar amyloid in Alzheimer’s disease. Eur. J. Nucl. Med. Mol. Imaging.

[7] Okello A (2009). Conversion of amyloid positive and negative MCI to AD over 3 years: an 11C-PIB PET study. Neurology.

[8] Ostrowitzki S (2017). A phase III randomized trial of gantenerumab in prodromal Alzheimer’s disease. Alzheimers Res. Ther..

[9] Roe CM (2013). Amyloid imaging and CSF biomarkers in predicting cognitive impairment up to 7.5 years later. Neurology.

[10] Buchhave P (2012). Cerebrospinal fluid levels of beta-amyloid 1-42, but not of tau, are fully changed already 5 to 10 years before the onset of Alzheimer dementia. Arch. Gen. Psychiatry.

[11] Hansson O (2006). Association between CSF biomarkers and incipient Alzheimer’s disease in patients with mild cognitive impairment: a follow-up study. Lancet Neurol..

[12] Hertze J (2010). Evaluation of CSF biomarkers as predictors of Alzheimer’s disease: a clinical follow-up study of 4.7 years. J. Alzheimers Dis..

[13] Mattsson N (2009). CSF biomarkers and incipient Alzheimer disease in patients with mild cognitive impairment. JAMA.

[14] Shaw LM (2009). Cerebrospinal fluid biomarker signature in Alzheimer’s disease neuroimaging initiative subjects. Ann. Neurol..

[15] Visser PJ (2009). Prevalence and prognostic value of CSF markers of Alzheimer’s disease pathology in patients with subjective cognitive impairment or mild cognitive impairment in the DESCRIPA study: a prospective cohort study. Lancet Neurol..

[16] Bittner T (2016). Technical performance of a novel, fully automated electrochemiluminescence immunoassay for the quantitation of beta-amyloid (1-42) in human cerebrospinal fluid. Alzheimers Dement..

[17] Hansson O (2018). CSF biomarkers of Alzheimer’s disease concord with amyloid-beta PET and predict clinical progression: a study of fully automated immunoassays in BioFINDER and ADNI cohorts. Alzheimers Dement..

[18] Mattsson, N. *et al*. CSF biomarker variability in the Alzheimer’s Association quality control program [published correction appears in *Alzheimers Dement*. 11, 237 (2015)]. *Alzheimers Dement*. 9, 251–261 (2013). 10.1016/j.jalz.2013.01.010 PMC3707386 23622690

[19] Lifke V (2019). Elecsys Total-Tau and Phospho-Tau (181P) CSF assays: Analytical performance of the novel, fully automated immunoassays for quantification of tau proteins in human cerebrospinal fluid. Clin Biochem..

[20] Shaw LM (2017). Deriving a cut-off for the Elecsys β-amyloid (1–42) immunoassay for use in clinical trials supported by Eli Lilly for patients with clinically defined Alzheimer’s disease (AD). Alzheimers Dement..

[21] Aisen PS, Petersen RC, Donohue M, Weiner MW (2015). ADNI 2 clinical core: progress and plans. Alzheimers Dement..

[22] Manjer J (2001). The M Diet and Cancer Study: representativity, cancer incidence and mortality in participants and non-participants. Eur. J. Cancer Prev..

[23] Mattsson N (2016). Increased amyloidogenic APP processing in APOE varepsilon4-negative individuals with cerebral beta-amyloidosis. Nat. Commun..

[24] American Psychiatric Association. *Diagnostic and Statistical Manual of Mental Disorders*. 3rd ed. Text revision (American Psychiatric Association, 1987).

[25] McKhann G (1984). Clinical diagnosis of Alzheimer’s disease: report of the NINCDS-ADRDA Work Group under the auspices of Department of Health and Human Services Task Force on Alzheimer’s Disease. Neurology.

[26] Blennow K, Hampel H, Weiner M, Zetterberg H (2010). Cerebrospinal fluid and plasma biomarkers in Alzheimer disease. Nat. Rev. Neurol..

[27] Blennow K, Zetterberg H, Fagan AM (2012). Fluid biomarkers in Alzheimer disease. Cold Spring Harb. Perspect. Med..

[28] Ferreira D (2014). Improving CSF biomarkers’ performance for predicting progression from mild cognitive impairment to Alzheimer’s disease by considering different confounding factors: a meta-analysis. Front. Aging Neurosci..

[29] Scheltens P (2016). Alzheimer’s disease. Lancet.

[30] Wang J, Xia Y, Grundke-Iqbal I, Iqbal K (2012). Abnormal hyperphosphorylation of tau: sites, regulation and molecular mechanism of neurofibrillary degeneration. J. Alzheimers Dis..

[31] Janelidze S (2017). Concordance between different amyloid immunoassays and visual amyloid positron emission tomographic assessment. JAMA Neurol..

[32] Schindler SE (2018). Cerebrospinal fluid biomarkers measured by Elecsys assays compared to amyloid imaging. Alzheimers Dement..

[33] Seeburger JL (2015). Cerebrospinal fluid biomarkers distinguish postmortem-confirmed Alzheimer’s disease from other dementias and healthy controls in the OPTIMA cohort. J. Alzheimers Dis..

[34] Jack CR (2016). A/T/N: an unbiased descriptive classification scheme for Alzheimer disease biomarkers. Neurology.

[35] Lewczuk P (2015). Validation of the Erlangen Score Algorithm for the prediction of the development of dementia due to Alzheimer’s disease in pre-dementia subjects. J. Alzheimers Dis..

[36] Jicha GA (2006). Neuropathologic outcome of mild cognitive impairment following progression to clinical dementia. Arch. Neurol..

[37] Cairns NJ (2015). Neuropathologic assessment of participants in two multi-center longitudinal observational studies: the Alzheimer Disease Neuroimaging Initiative (ADNI) and the Dominantly Inherited Alzheimer Network (DIAN). Neuropathology.

[38] Toledo JB (2013). Clinical and multimodal biomarker correlates of ADNI neuropathological findings. Acta Neuropathol. Commun..

[39] O’Bryant SE (2017). Blood-based biomarkers in Alzheimer disease: Current state of the science and a novel collaborative paradigm for advancing from discovery to clinic. Alzheimers Dement..

[40] Hampel H (2018). Blood-based biomarkers for Alzheimer’s disease: mapping the road to the clinic. Nature Rev Neurol..

[41] Frolich L (2017). Incremental value of biomarker combinations to predict progression of mild cognitive impairment to Alzheimer’s dementia. Alzheimers Res. Ther..

