# Supplementary material for: Predicting clinical decline and conversion to Alzheimer’s disease or dementia using novel Elecsys Aβ(1–42), pTau and tTau CSF immunoassays
Source: Sci Rep. 2019 Dec 13;9:19024. doi: 10.1038/s41598-019-54204-z (PMC6911086; doi:10.1038/s41598-019-54204-z)
Supplement: Supplementary file 1 — Supplement [file 41598_2019_54204_MOESM1_ESM.pdf]

Supplementary Materials

Predicting clinical decline and conversion to Alzheimer's disease or dementia using novel Elecsys Aβ(1–42), pTau and tTau CSF immunoassays

Kaj Blennow[1,2,†], Leslie M. Shaw[3,†], Erik Stomrud[4,5], Niklas Mattsson[4,6], Jon B. Toledo[3,7], Katharina Buck[8,‡], Simone Wahl[8,‡], Udo Eichenlaub[8], Valeria Lifke[8], Maryline Simon[9], John Q. Trojanowski[3] & Oskar Hansson[4,5,*]

[1]Clinical Neurochemistry Laboratory, Sahlgrenska University Hospital, Mölndal, Sweden. [2]Institute of Neuroscience and Physiology, Department of Psychiatry and Neurochemistry, The Sahlgrenska Academy at University of Gothenburg, Mölndal, Sweden. [3]Department of Pathology and Laboratory Medicine, University of Pennsylvania, Philadelphia, PA, USA. [4]Clinical Memory Research Unit, Lund University, Malmö, Sweden. [5]Memory Clinic, Skåne University Hospital, Malmö, Sweden. [6]Wallenberg Center for Molecular Medicine, Lund University, Lund, Sweden. [7]Department of Neurology, Houston Methodist Hospital, Houston, TX, USA. [8]Roche Diagnostics GmbH, Penzberg, Germany. [9]Roche Diagnostics International Ltd, Rotkreuz, Switzerland.

[†]These authors contributed equally to the manuscript.

[‡]These authors completed the statistical analysis.

***Corresponding author:** Oskar Hansson

Address: Clinical Memory Research Unit, Lund University, Sweden

Email: oskar.hansson@med.lu.se

Telephone: +46 72 226 77 45

Fax: +46 (0)46 222 4720

Tables

Supplementary Table S1. Prediction of clinical decline assessed by model estimates for all covariates from baseline to 24 months according to the mixed-model of pTau/Aβ(1–42) and outcome MMSE in the ADNI MCI and BioFINDER MCS populations.

Study	Effect	Estimate	P value
ADNI	Intercept	0.42	0.7731
	Visit (6 months)	9.38	<0.0001
	Visit (12 months)	8.69	<0.0001
	Visit (24 months)	6.52	<0.0001
	BM+	0.02	0.8823
	Visit (6 months)*BM+	–1.00	<0.0001
	Visit (6 months)*BM–	0.00	
	Visit (12 months)*BM+	–1.39	<0.0001
	Visit (12 months)*BM–	0.00	
	Visit (24 months)*BM+	–2.08	<0.0001
	Visit (24 months)*BM–	0.00	
	Age	–0.01	0.2322
	Gender (female)	–0.09	0.4398
	Education	0.04	0.0454
	MMSE_baseline*Visit (0 months)	0.99	<0.0001
	MMSE_baseline*Visit (6 months)	0.65	<0.0001
	MMSE_baseline*Visit (12 months)	0.68	<0.0001
	MMSE_baseline*Visit (24 months)	0.75	<0.0001
BioFINDER	Intercept	0.32	0.8751
	Visit (12 months)	7.60	0.0006
	Visit (24 months)	1.09	0.6308
	BM+	0.00	0.9941
	Visit (12 months)*BM+	–1.23	<0.0001
	Visit (12 months)*BM–	0.00	
	Visit (24 months)*BM+	–1.57	<0.0001
	Visit (24 months)*BM–	0.00	
	Age	0.00	0.7723
	Gender (female)	0.12	0.4276
	Education	0.02	0.3706
	MMSE_baseline	0.99	<0.0001

	MMSE_baseline*Visit (12 months)	–0.28	0.0003
	MMSE_baseline*Visit (24 months)	–0.07	0.4097

Analyses shown with adjustment for age, sex, years of education but without adjustment for APOEε4 status. pTau, phosphorylated Tau; MMSE, Mini-Mental State Examination; ADNI, Alzheimer's Disease Neuroimaging Initiative; MCI, mild cognitive impairment; MCS, mild cognitive symptoms; BM+, biomarker-positive; BM–, biomarker-negative.

Supplementary Table S2. Prediction of clinical decline assessed by change in clinical scores in CDR-SB (ADNI only), FAQ and ADAS-cog (ADNI only) from baseline to 24 months according to CSF biomarker status, in the ADNI MCI and BioFINDER MCS populations.

Clinical score	Biomarker	Change in score, BM-positive, estimate (95% CI)		Change in score, BM-negative, estimate (95% CI)		Difference between change in score, BM-negative and BM-positive, estimate (95% CI)	
		ADNI	BioFINDER	ADNI	BioFINDER	ADNI	BioFINDER
**CDR-SB**	pTau/Aβ(1–42)	1.50 (1.36 to 1.64)		0.16 (0.01 to 0.31)		1.34 (1.13 to 1.55)	
	tTau/Aβ(1–42)	1.51 (1.37 to 1.65)		0.14 (–0.01 to 0.30)		1.37 (1.16 to 1.58)	
	Aβ(1–42)	1.36 (1.23 to 1.49)		0.19 (0.03 to 0.36)		1.17 (0.95 to 1.38)	
	pTau	1.47 (1.31 to 1.63)		0.48 (0.34 to 0.62)		1.00 (0.78 to 1.21)	
	tTau	1.46 (1.29 to 1.64)		0.56 (0.43 to 0.69)		0.90 (0.68 to 1.12)	

**FAQ**	pTau/Aβ(1–42)	4.54 (4.08 to 4.99)	4.83 (4.01 to 5.66)	0.84 (0.34 to 1.34)	2.31 (1.59 to 3.03)	3.70 (3.01 to 4.38)	2.52 (1.42 to 3.62)
	tTau/Aβ(1–42)	4.56 (4.11 to 5.01)	4.83 (4.01 to 5.66)	0.79 (0.28 to 1.29)	2.31 (1.59 to 3.03)	3.78 (3.09 to 4.46)	2.52 (1.42 to 3.62)
	Aβ(1–42)	4.25 (3.81 to 4.68)	4.35 (3.58 to 5.11)	0.80 (0.27 to 1.33)	2.43 (1.65 to 3.21)	3.45 (2.75 to 4.14)	1.92 (0.82 to 3.01)
	pTau	4.57 (4.05 to 5.09)	4.19 (3.19 to 5.20)	1.63 (1.19 to 2.07)	3.07 (2.41 to 3.72)	2.94 (2.25 to 3.63)	1.13 (–0.07 to 2.33)
	tTau	4.69 (4.14 to 5.24)	4.40 (3.42 to 5.38)	1.78 (1.35 to 2.20)	2.95 (2.29 to 3.61)	2.91 (2.21 to 3.60)	1.45 (0.26 to 2.63)
**ADAS-cog**	pTau/Aβ(1–42)	3.98 (3.37 to 4.58)		–0.12 (–0.82 to 0.58)		4.09 (3.13 to 5.06)	
	tTau/Aβ(1–42)	3.99 (3.39 to 4.60)		–0.17 (–0.87 to 0.53)		4.16 (3.20 to 5.12)	

	Aβ(1–42)	3.54 (2.97 to 4.12)		0.04 (–0.70 to 0.79)		3.50 (2.53 to 4.47)	
	pTau	4.00 (3.31 to 4.69)		0.81 (0.21 to 1.41)		3.19 (2.24 to 4.13)	
	tTau	3.79 (3.06 to 4.52)		1.22 (0.65 to 1.79)		2.57 (1.62 to 3.52)	

Based on PET-optimised cut-offs for Aβ(1–42), pTau/Aβ(1–42) and tTau/Aβ(1–42). Analyses shown with adjustment for age, sex, years of education but without adjustment for *APOE*ε4 status. CDR-SB, Clinical Dementia Rating Scale Sum of Boxes; ADNI, Alzheimer's Disease Neuroimaging Initiative; FAQ, Functional Activities Questionnaire; ADAS-cog, Alzheimer’s Disease Assessment Scale-cognitive; CSF, cerebrospinal fluid; MCI, mild cognitive impairment; MCS, mild cognitive symptoms; BM, biomarker; CI, confidence interval; pTau, phosphorylated Tau; tTau, total Tau; PET, positron emission tomography.

Supplementary Table S3. Prediction of clinical decline assessed by change in MMSE, CDR-SB, FAQ and ADAS-cog clinical scores from baseline to 24 months according to CSF biomarker status, in the ADNI MCI population.

Clinical score	Biomarker	Change in score, BM-positive, estimate (95% CI)	Change in score, BM-negative, estimate (95% CI)	Difference between change in score, BM-negative and BM-positive, estimate (95% CI)
**MMSE**	pTau/Aβ(1–42)	–2.25 (–2.51 to –1.98)	–0.08 (–0.36 to 0.20)	–2.17 (–2.56 to –1.77)
	tTau/Aβ(1–42)	–2.32 (–2.59 to –2.04)	–0.13 (–0.40 to 0.14)	–2.19 (–2.58 to –1.79)
	Aβ(1–42)	–2.04 (–2.30 to –1.78)	–0.25 (–0.53 to 0.04)	–1.79 (–2.19 to –1.40)
**CDR-SB**	pTau/Aβ(1–42)	1.59 (1.44 to 1.73)	0.17 (0.02 to 0.32)	1.42 (1.21 to 1.62)
	tTau/Aβ(1–42)	1.62 (1.47 to 1.76)	0.21 (0.07 to 0.35)	1.41 (1.20 to 1.62)
	Aβ(1–42)	1.41 (1.27 to 1.55)	0.31 (0.16 to 0.46)	1.10 (0.89 to 1.31)
**FAQ**	pTau/Aβ(1–42)	4.80 (4.33 to 5.26)	0.84 (0.36 to 1.32)	3.96 (3.28 to 4.64)
	tTau/Aβ(1–42)	4.94 (4.46 to 5.41)	0.93 (0.46 to 1.39)	4.01 (3.33 to 4.69)
	Aβ(1–42)	4.46 (4.00 to 4.92)	1.09 (0.60 to 1.57)	3.37 (2.70 to 4.05)
**ADAS-cog**	pTau/Aβ(1–42)	4.20 (3.57 to 4.84)	–0.08 (–0.76 to 0.60)	4.28 (3.30 to 5.26)
	tTau/Aβ(1–42)	4.47 (3.83 to 5.12)	–0.09 (–0.75 to 0.56)	4.57 (3.60 to 5.53)
	Aβ(1–42)	3.72 (3.11 to 4.33)	0.39 (–0.29 to 1.06)	3.33 (2.39 to 4.27)

Based on pre-specified cut-offs adjusted for pre-analytical handling: Aβ(1–42), 880 pg/mL; pTau/Aβ(1–42), 0.028; tTau/Aβ(1–42), 0.33. Analyses shown with adjustment for age, sex, years of education but without adjustment for *APOE*ε4 status. MMSE, Mini-Mental State Examination; CDR-SB, Clinical Dementia Rating Scale Sum of Boxes; FAQ, Functional Activities Questionnaire; ADAS-cog, Alzheimer's Disease Assessment Scale-cognitive; CSF, cerebrospinal fluid; ADNI, Alzheimer's Disease Neuroimaging Initiative; MCI, mild cognitive impairment; BM, biomarker; CI, confidence interval; pTau, phosphorylated Tau; tTau, total Tau.

Figures

Supplementary Figure S1. Difference of model-derived least square-means (with 95% CI) in MMSE score change from baseline to 24 months according to CSF biomarker status, in the ADNI MCI and BioFINDER MCS populations.

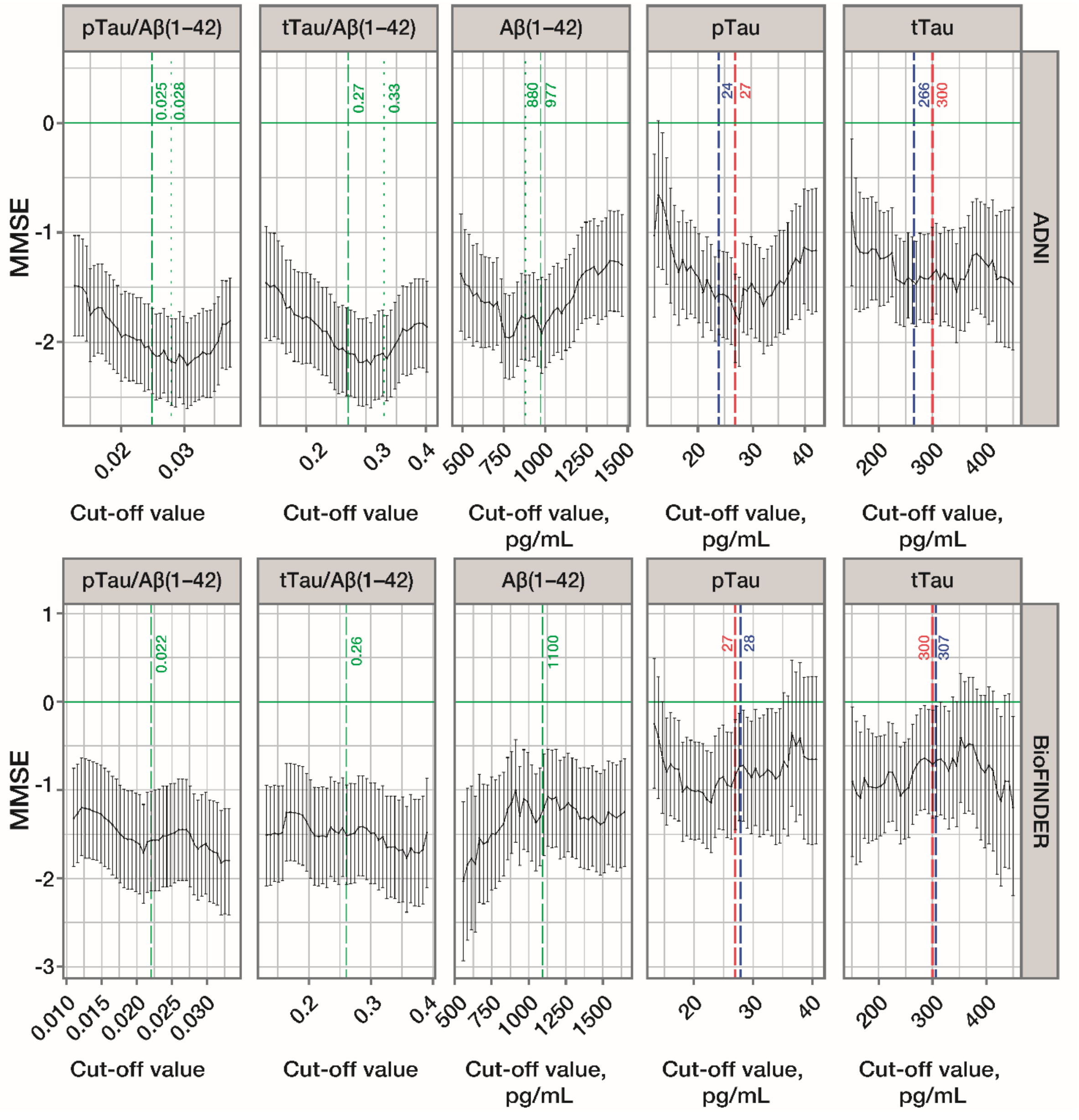

Vertical lines represent the cut-offs (dashed, optimised in respective cohort; dotted, pre-specified; green, optimised for PET concordance; red, determined using progression data; blue, optimised for AD *versus* controls); ADNI (upper panel) and BioFINDER (lower panel). Analyses shown with adjustment for age, sex, years of education but without adjustment for *APOE*ε4 status. CI, confidence interval; MMSE, Mini-Mental State Examination; CSF, cerebrospinal fluid; ADNI, Alzheimer's Disease Neuroimaging Initiative; MCI, mild cognitive impairment; MCS, mild cognitive symptoms; pTau, phosphorylated Tau; tTau, total Tau; PET, positron emission tomography; AD, Alzheimer's disease.

Supplementary Figure S2. Difference of least square-means in change in (**a**) CDR-SB (ADNI MCI only), (**b**) FAQ (ADNI MCI and BioFINDER MCS) clinical scores from baseline to 24 months according to CSF biomarker status.

a

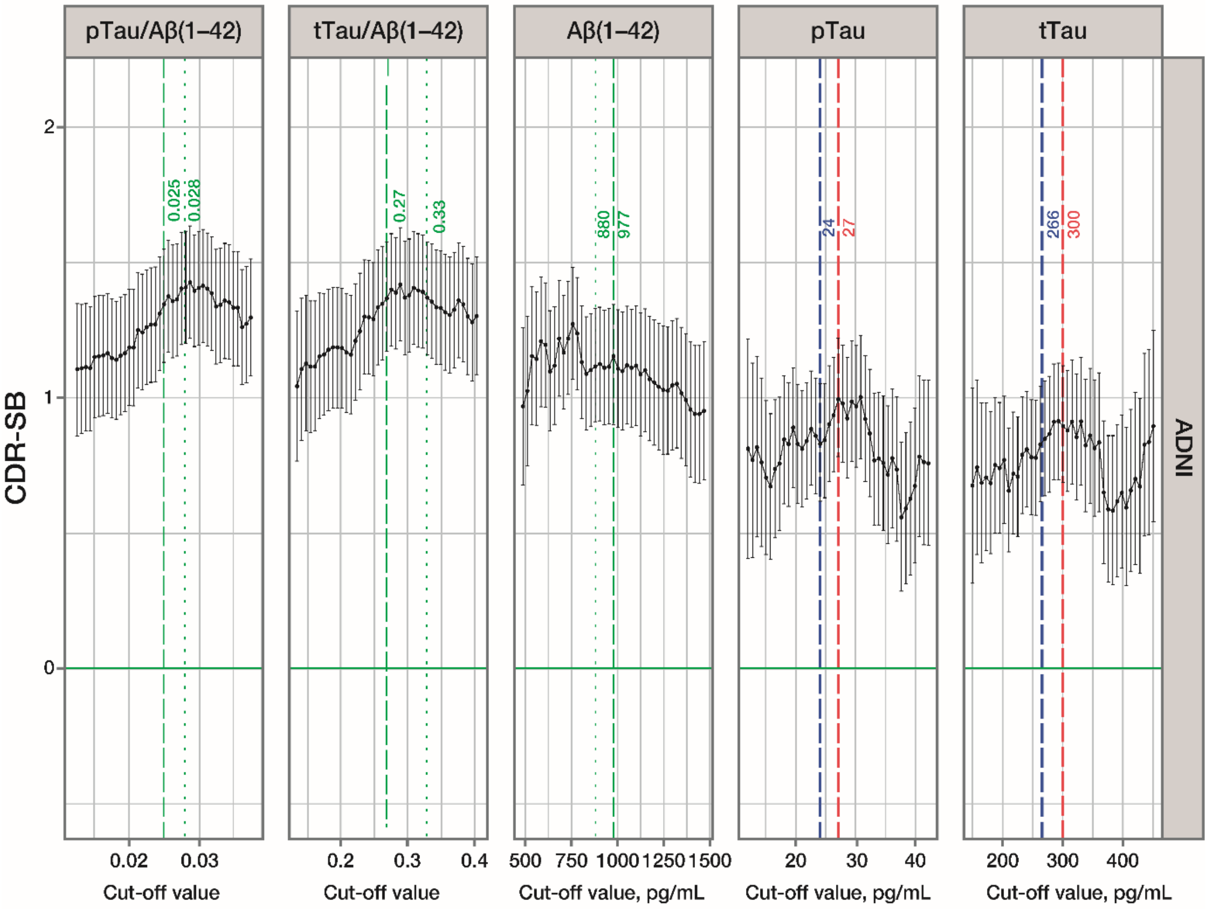

b

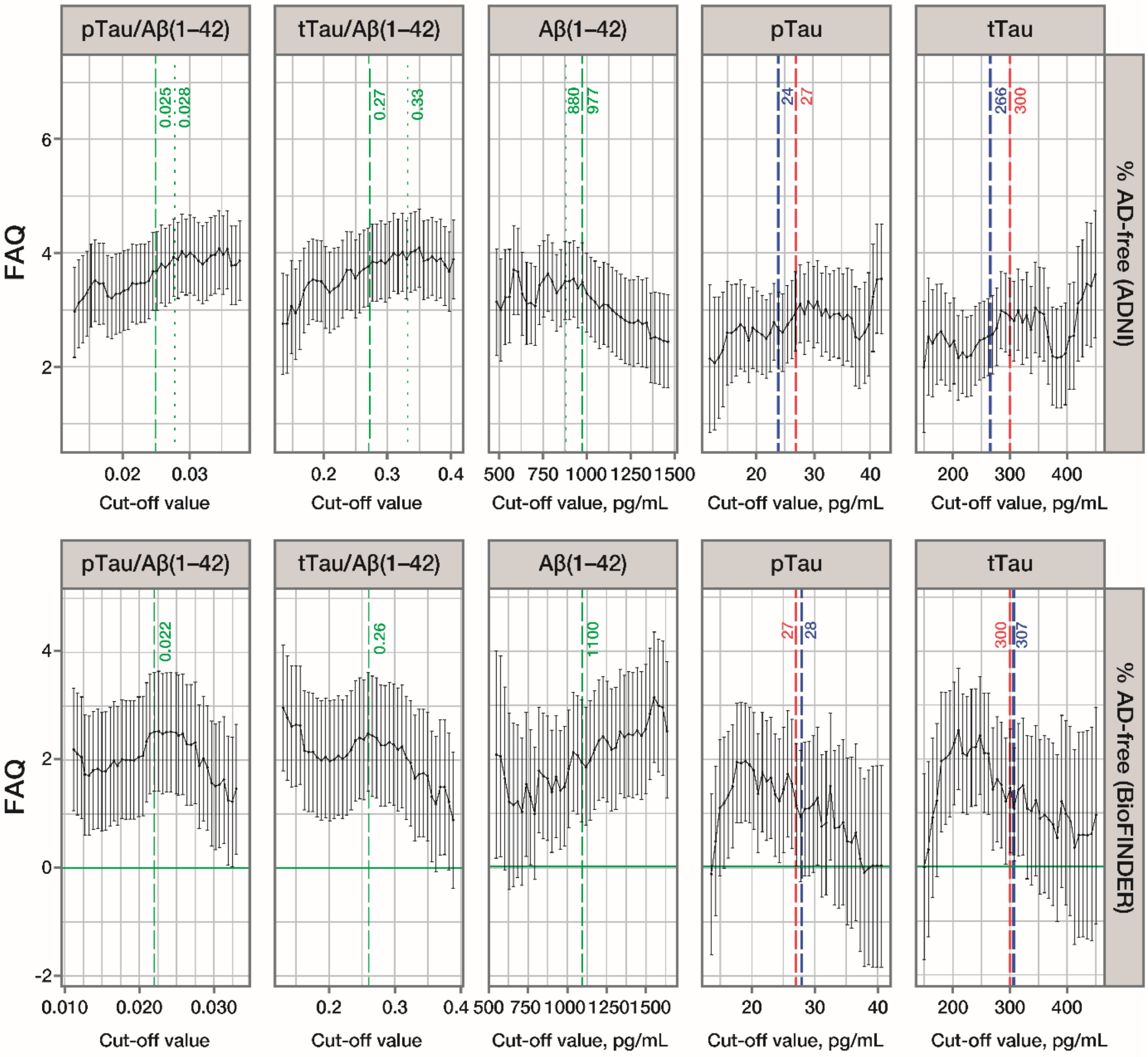

Vertical lines represent the cut-offs (dashed: optimised in respective cohort; dotted: pre-specified; green: optimised for PET concordance; red: determined using progression data; blue: optimised for AD *versus* controls). Analyses shown with adjustment for age, sex, years of education but without adjustment for *APOE*ε4 status. CDR-SB, Clinical Dementia Rating Sum of Boxes; ADNI, Alzheimer's Disease Neuroimaging Initiative; MCI, mild cognitive impairment; FAQ, Functional Activities Questionnaire; MCS, mild cognitive symptoms; CSF, cerebrospinal fluid; pTau, phosphorylated Tau; tTau, total Tau; AD, Alzheimer’s disease; PET, positron emission tomography.

Supplementary Figure S3. Cut-off grid derived hazard ratios (with 95% CIs) for outcome time to dementia according to CSF biomarkers (pTau/Aβ(1–42), tTau/Aβ(1–42), Aβ(1–42), pTau, tTau), in the ADNI MCI (upper panel) and BioFINDER MCS (lower panel) populations.

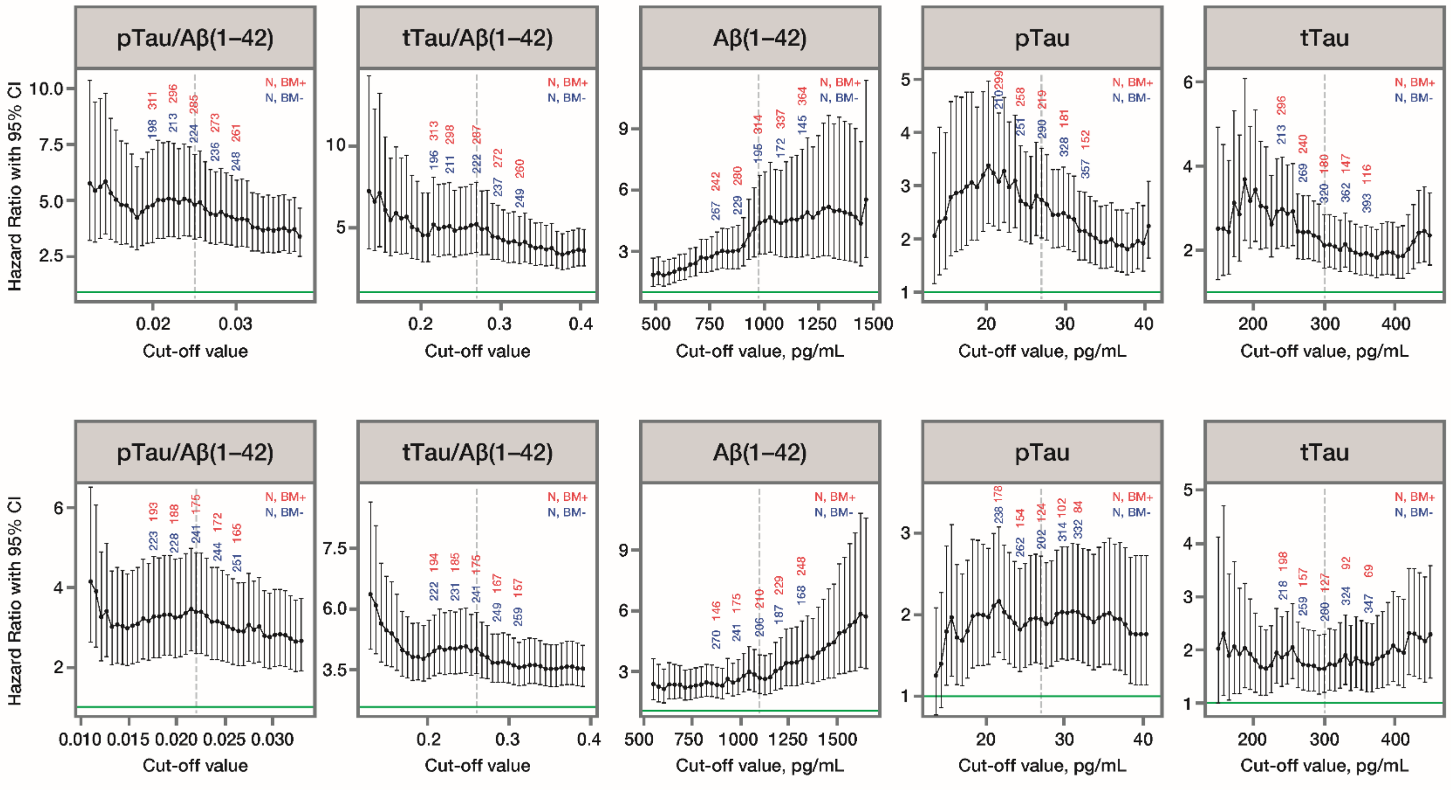

Analyses shown with adjustment for age, sex, years of education, baseline MMSE score, baseline CDR-SB score (ADNI only), but without adjustment for *APOE*ε4 status. CI, confidence interval; CSF, cerebrospinal fluid; pTau, phosphorylated Tau; tTau, total Tau; ADNI, Alzheimer's Disease Neuroimaging Initiative; MCI, mild cognitive impairment; MCS, mild cognitive symptoms; BM+, biomarker-positive; BM–, biomarker-negative; MMSE, Mini-Mental State Examination; CDR-SB, Clinical Dementia Rating Sum of Boxes.

Supplementary Figure S4. Model-derived time-course plots (least square-means with SEs) of (**a**) FAQ (ADNI MCI and BioFINDER MCS populations), (**b**) CDR-SB (ADNI MCI only) and (**c**) ADAS-cog (ADNI MCI only) clinical scores from baseline to 24 months according to CSF biomarker status; number of patients in each biomarker group at baseline is presented.

a

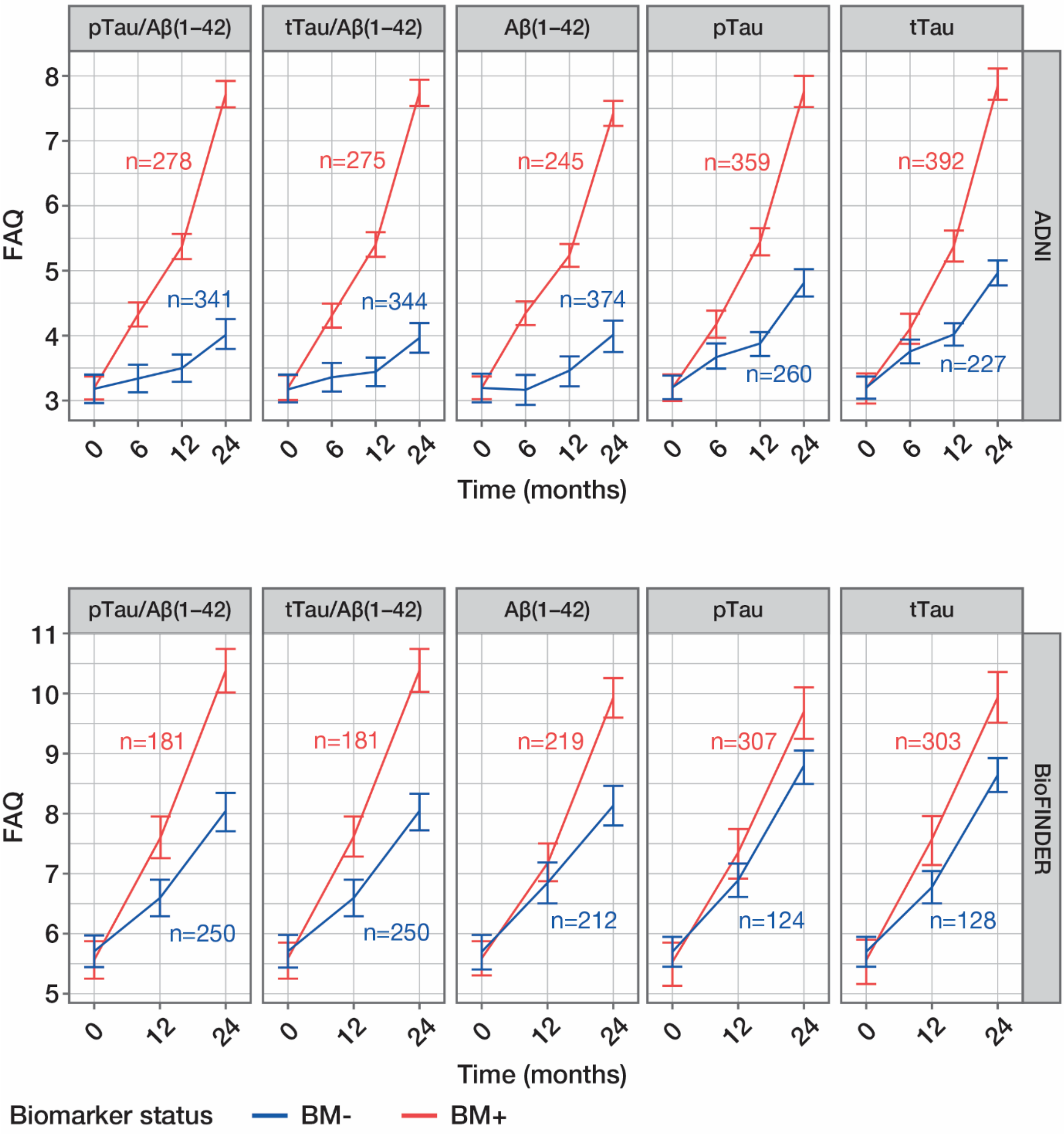

b

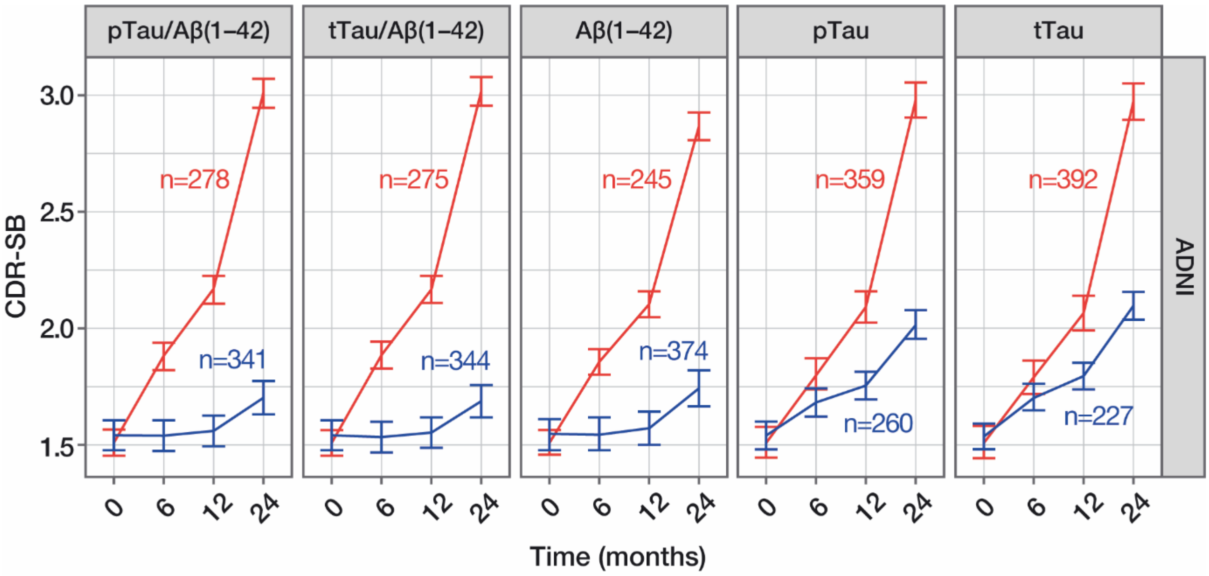

c

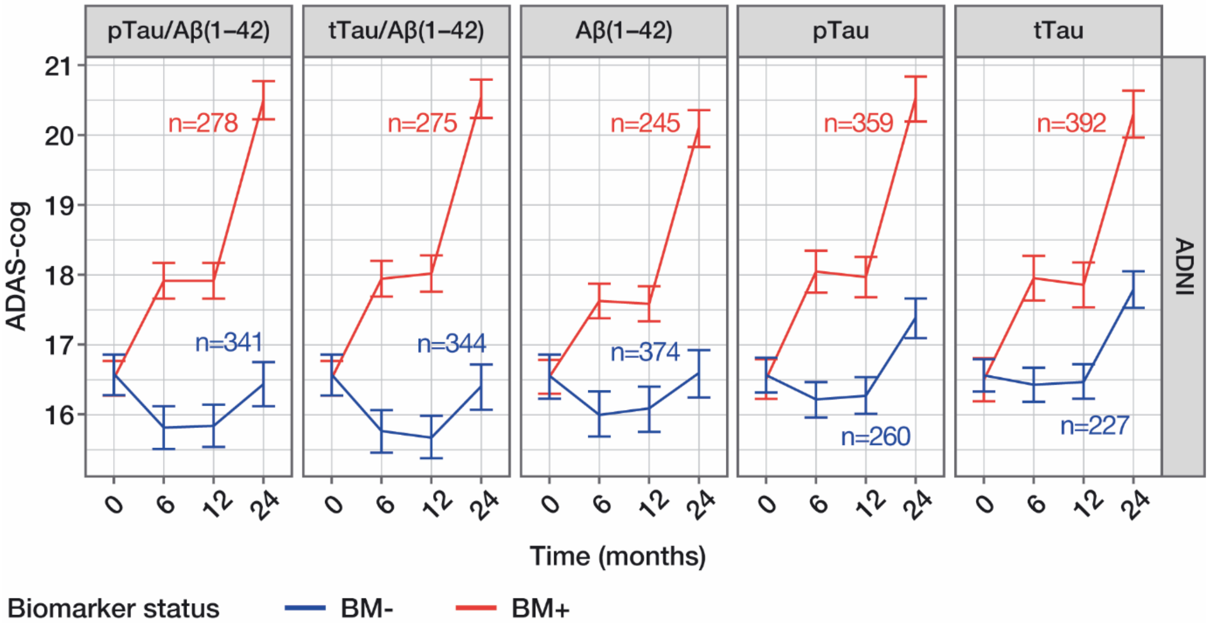

Based on PET-optimised cut-offs for Aβ(1–42), pTau/Aβ(1–42) and tTau/Aβ(1–42). Analyses shown with adjustment for age, sex, years of education but without adjustment for *APOE*ε4 status. SE, standard error; FAQ, Functional Activities Questionnaire; ADNI, Alzheimer's Disease Neuroimaging Initiative; MCI, mild cognitive impairment; MCS, mild cognitive symptoms; CDR-SB, Clinical Dementia Rating Sum of Boxes; ADAS-cog, Alzheimer’s Disease Assessment Scale-cognitive; CSF, cerebrospinal fluid; pTau, phosphorylated Tau; tTau, total Tau; BM –, biomarker-negative; BM+, biomarker-positive; PET, positron emission tomography.

Supplementary Figure S5. Model-derived time-course plots (least square-means with SEs) of MMSE score from baseline to 24 months according to biomarker status, in the ADNI MCI population; number of patients in each biomarker group at baseline is presented.

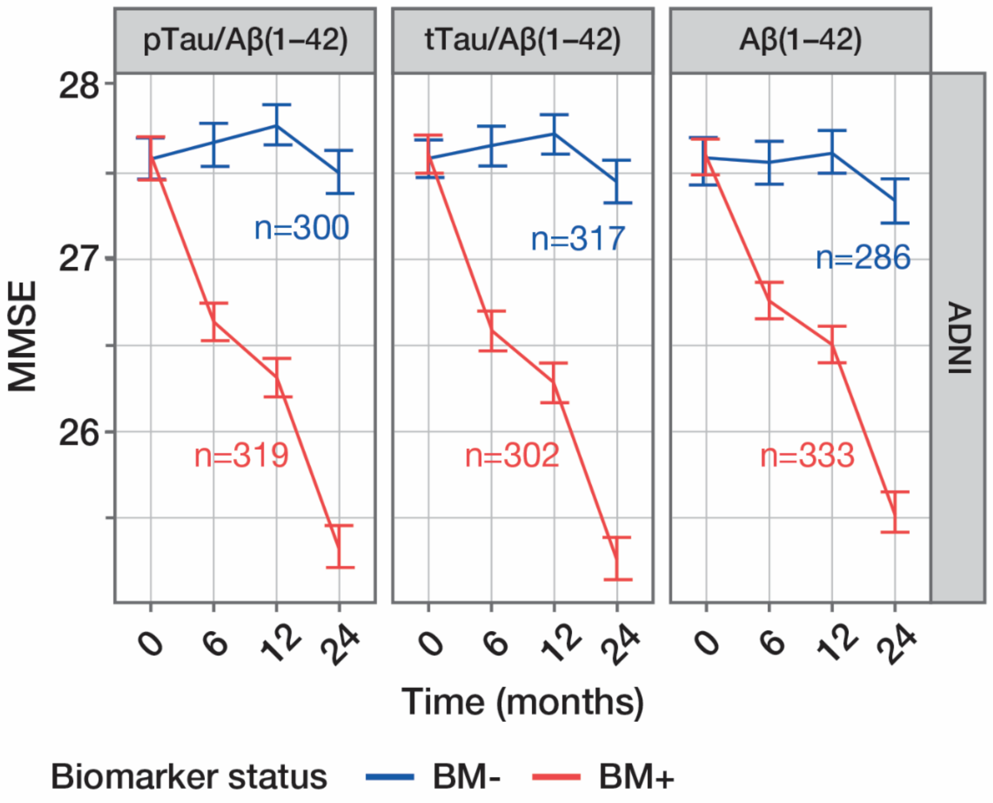

Based on pre-specified, adjusted cut-offs for Aβ(1–42), 880 pg/mL; pTau/Aβ(1–42), 0.028; tTau/Aβ(1–42), 0.33. SE, standard error; MMSE, Mini-Mental State Examination; ADNI, Alzheimer's Disease Neuroimaging Initiative; MCI, minimal cognitive impairment; pTau, phosphorylated Tau; tTau, total Tau; BM–, biomarker-negative; BM+, biomarker-positive.

Supplementary Figure S6. Model-derived time-course plots (least square-means with SE) of MMSE score from baseline to 24 months according to pTau and pTau/Aβ(1–42) status in the (**a**) ADNI MCI and (**c**) BioFINDER MCS populations; number of patients in each biomarker group at baseline is presented. Kaplan-Meier plots of outcome dementia diagnosis within 6 years of follow-up according to pTau and pTau/Aβ(1–42) status in the (**b**) ADNI MCI and (**d**) BioFINDER MCS populations; number of patients in each biomarker group at each time point is presented.

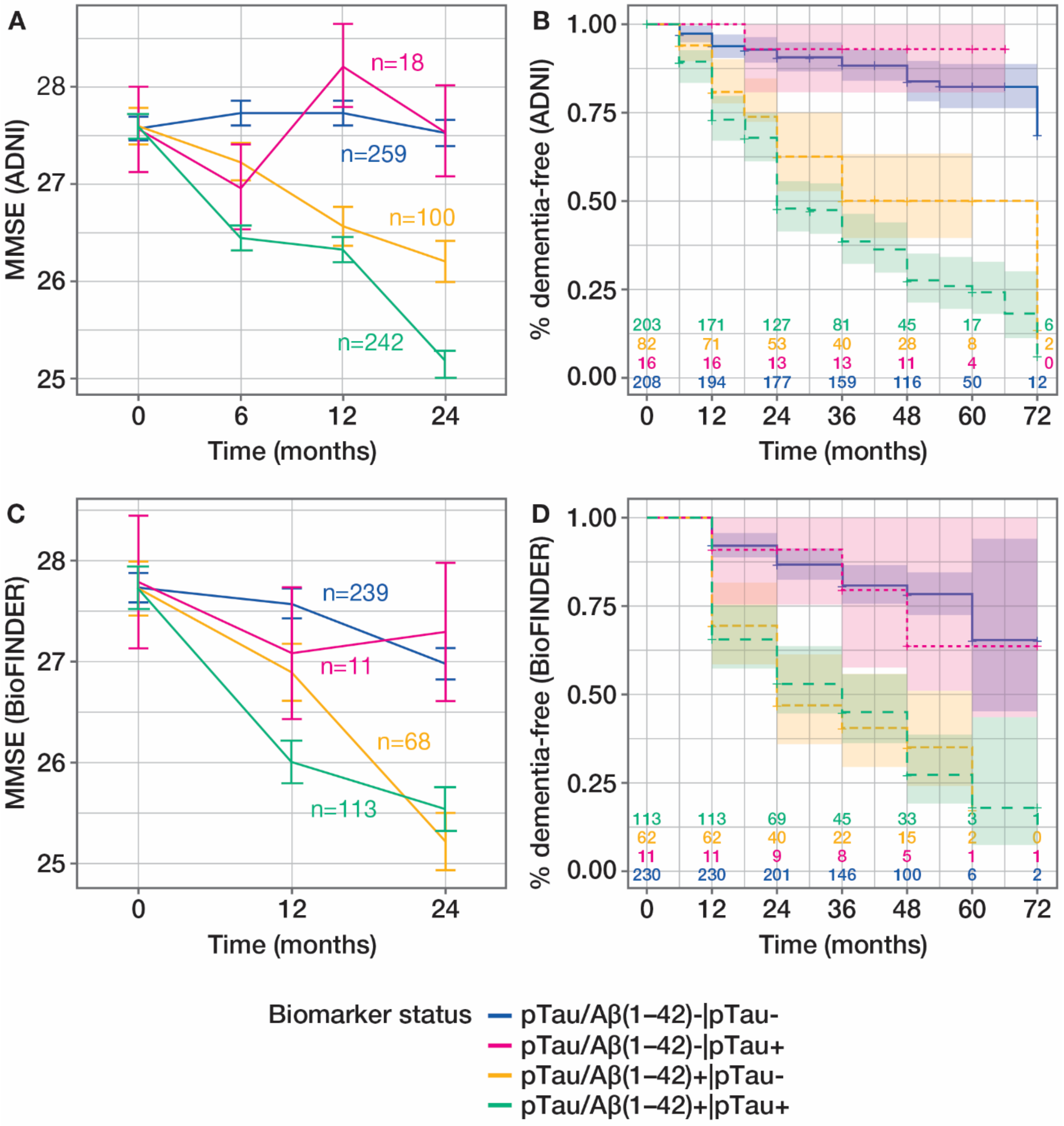

Mixed-model analyses (**a**, **c**) shown with adjustment for age, sex, years of education but without adjustment for *APOEε4* status. SE, standard error; MMSE, Mini-Mental State Examination; pTau, phosphorylated Tau; ADNI, Alzheimer's Disease Neuroimaging Initiative; MCI, mild cognitive impairment; MCS, mild cognitive symptoms.

Supplementary Figure S7. Model-derived time-course plots (least square-means with SE) of MMSE score from baseline to 24 months according to pTau and visual PET status in the (**a**) ADNI MCI and (**c**) BioFINDER MCS populations; number of patients in each biomarker group at baseline is presented. Kaplan-Meier plots of outcome dementia diagnosis within 6 years of follow-up according to pTau and visual PET status in the (**b**) ADNI MCI and (**d**) BioFINDER MCS populations; number of patients in each biomarker group at each time point is presented.

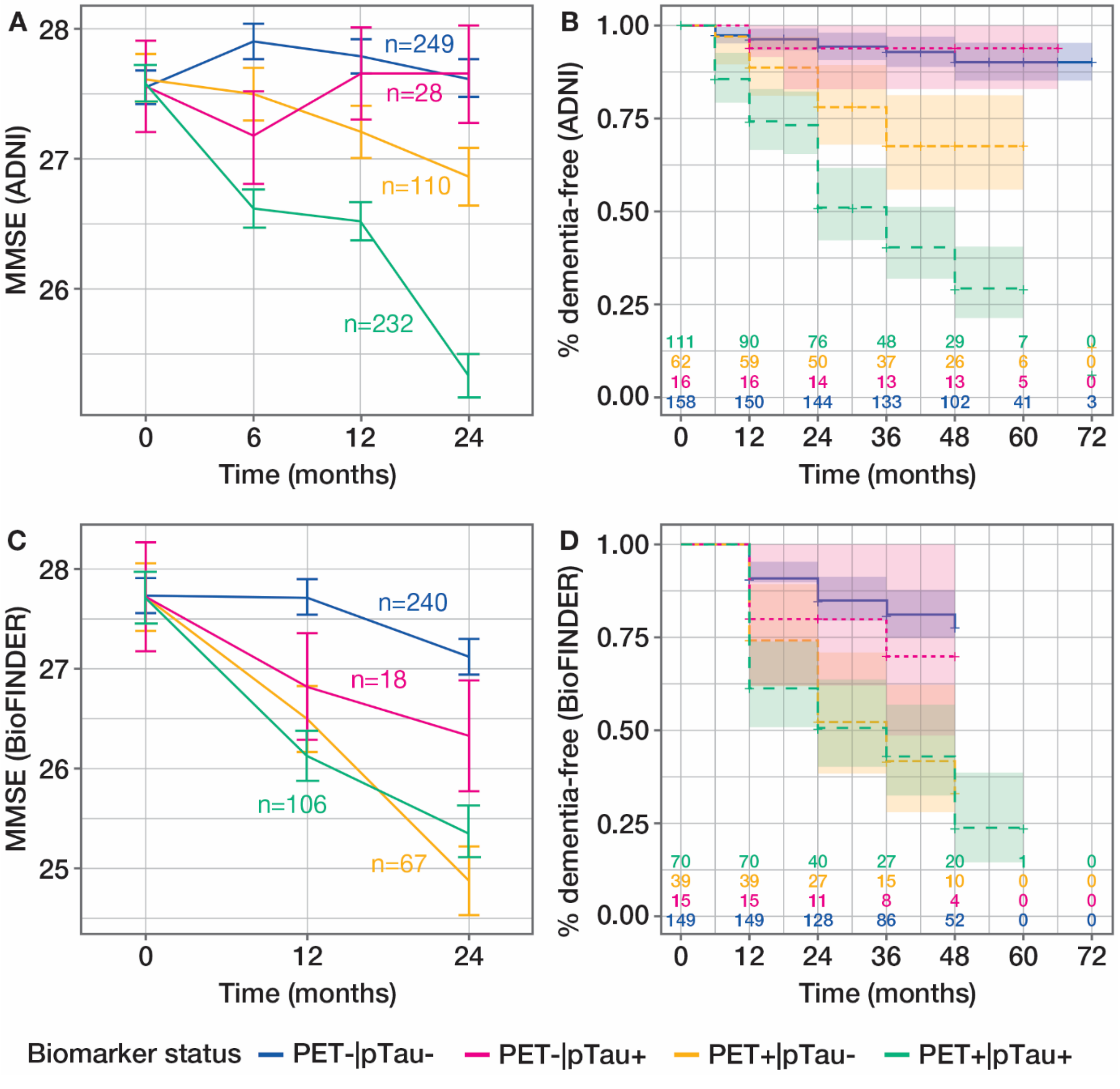

Mixed-model analyses (**a**, **c**) shown with adjustment for age, sex, years of education but without adjustment for *APOE*ε4 status. SE, standard error; MMSE, Mini-Mental State Examination; pTau, phosphorylated Tau; PET, positron emission tomography; ADNI, Alzheimer's Disease Neuroimaging Initiative; MCI, mild cognitive impairment; MCS, mild cognitive symptoms.
